# Epidemiological pattern and incidence estimation of infectious diarrhea before the COVID-19 pandemic: Beijing, China, 2015–2019

**DOI:** 10.3389/fpubh.2026.1717990

**Published:** 2026-01-21

**Authors:** Ruihan Xu, Yi Tian, Lei Jia, Gemixi Dedema, Jueqiong Zhao, Mei Qu, Weihong Li, Bing Lv, Xin Zhang, Ying Huang, Zhaomin Feng, Quanyi Wang, Zhiyong Gao, Daitao Zhang

**Affiliations:** 1 School of Public Health, China Medical University, Shenyang, China; 2 General Administration of Customs (Beijing) International Travel Health Care Center, Beijing, China; 3 Beijing Key Laboratory of Surveillance, Early Warning and Pathogen Research on Emerging Infectious Diseases, Beijing Center for Disease Prevention and Control, Beijing, China; 4 School of Public Health, Capital Medical University, Beijing, China; 5 Vanke School of Public Health, Tsinghua University, Beijing, China

**Keywords:** diarrhea, epidemiological characteristics, outpatients, pathogen, surveillance

## Abstract

**Objectives:**

To analyze the epidemiological characteristics and estimate the incidence of infectious diarrhea in Beijing, China, 2015–2019.

**Methods:**

From January 2015 to December 2019, stool specimens and epidemiological data of diarrhea cases were collected from sentinel hospitals across all districts. Viral pathogens were detected by real time PCR or RT-PCR, including rotavirus, norovirus, astrovirus, adenovirus and sapovirus. Bacterial pathogens were detected using culture, biochemical and serological assays, including *Vibrio cholerae*, Shigella, *Salmonella typhi* and *Salmonella paratyphi*, diarrheagenic *Escherichia coli* (DEC), non-typhoidal Salmonella (NTS), *Vibrio parahaemolyticus*, Campylobacter and other bacteria. Descriptive statistics and a Monte Carlo multiplier model were applied for analysis and incidence estimation.

**Results:**

A total of 27,804 outpatients were included from 2015 to 2019. Among cases tested for viral pathogens (*N* = 9,351), norovirus (1,572/9,351, 16.81%) was the most prevalent, followed by rotavirus (907/9,351, 9.70%). Among those tested for bacterial pathogens (*N* = 20,741), diarrheagenic *Escherichia coli* (DEC) (1,941/20,741, 9.36%) was predominant, followed by non-typhoidal Salmonella (NTS) (4.55%) and *Vibrio parahaemolyticus* (4.17%). Notably, enteroaggregative *E. coli* (35.65%) was the predominant type of DEC identified in this study. The detection rates of viral pathogens were higher in winter and spring. Rotavirus was mainly identified in cases under 5 years of age, while norovirus was more common in cases aged 18–40 years old. Bacterial pathogens were mainly identified in cases aged 18–40 years old in summer. Estimated by the model, the average annual number of infectious diarrhea cases in Beijing from 2015 to 2019 was approximately 68,054 (95%CI: 49,917–104,149). While the model result indicates the presence of significant hidden burden, but are constrained by inherent limitations in the modeling approach and monitoring design.

**Conclusion:**

Diarrhea remains a serious public health problem in Beijing, China. Norovirus, DEC, and rotavirus were the predominant pathogens among diarrhea outpatients in Beijing. Continuous surveillance is necessary for guiding prevention and control strategies.

## 1 Introduction

Diarrhea remains one of the important public health problems with a severe disease burden worldwide. In 2019, diarrhea caused 1.53 million deaths globally (1), and it was also one of the leading causes of disability-adjusted life years (DALYs) (2). Compared to before, although diarrhea-related mortality has been decreasing in recent years, the incidence rate has not decreased to a comparable degree (3, 4). About 836 million people suffer from diarrhea every year in China (5). The most common causes of diarrhea are infectious, including viruses, bacteria, and parasites, especially viruses and bacteria (6).

In order to identify the epidemiological and etiological characteristics of diarrhea comprehensively and timely, active surveillance of patients with diarrhea was conducted in 217 sentinel hospitals and 93 reference laboratories in 31 provinces (autonomous regions or municipalities) in mainland China from January 2009 to December 2018. Surveillance results showed that rotavirus A and norovirus were the most common viral pathogens, and diarrheagenic *Escherichia coli* (DEC) and non-typhoidal Salmonella (NTS) were the most common bacterial pathogens (7). The pathogen spectrum differed across age groups. Rotavirus is the most common cause of diarrhea in children under 5 years of age. However, the main pathogen of diarrhea in people over 70 years old is Shigella (2).

The epidemiological and etiological characteristics of diarrhea varied among different regions and years in China. Since 2011, Beijing has gradually established a viral diarrhea surveillance network. The system has gradually expanded from monitoring only children to all age groups. Detection of viral pathogens has also been expanded from two (rotavirus and norovirus) to five (rotavirus, norovirus, astrovirus, enteric adenovirus and sapovirus). Since 2012, Beijing has gradually established a bacterial diarrhea surveillance network in some districts. Until 2015, all 16 districts of Beijing had been included in the surveillance network. This study described the pathogen spectrum, detection rate and temporal changes of intestinal outpatients with diarrhea from 2015 to 2019 in Beijing.

However, the surveillance network mainly records cases that seek medical attention, making it difficult to determine the actual incidence in the population and the extent of under-reporting in Beijing. In order to close this gap, our work used a Monte Carlo multiplier model to estimate the overall community burden as well as evaluate the true incidence level of infectious diarrhea.

## 2 Materials and methods

### 2.1 Data source

Acute diarrhea is defined as three or more watery, loose, mucous or bloody stools within 24 h, lasting less than 14 days. Diarrhea cases due to inappropriate use of chemicals (such as diet pills) or taking antibiotics were excluded, as were cases who had resided in Beijing for less than 6 months.

Cases were collected from 2 surveillance networks from January 2015 to December 2019. For viral diarrhea surveillance, about 10 stool specimens were collected monthly from diarrhea cases at 1 or 2 sentinel hospitals in each of the 16 districts. Regarding bacterial diarrhea surveillance, about 40 stool specimens were collected from 1 or 2 sentinel hospitals in each of the 16 districts from April to October, and 10 stool specimens in the remaining months. In 2019, the two surveillance networks were integrated. The viral pathogen surveillance cases should be derived from the bacterial pathogen surveillance cases as much as possible. The monitoring results of viruses and bacteria were analyzed separately, and a comparative analysis was only conducted when the two systems were integrated in 2019.

All cases were patients at their first visit to the enteric outpatient clinics. Socio-demographic characteristics were recorded, including district, onset time, sex and age. Dongcheng, Xicheng, Chaoyang, Haidian, Fengtai, and Shijingshan districts were defined as urban areas, and the remaining districts were defined as rural areas.

### 2.2 Specimen collection and laboratory tests

For each included diarrhea case, about 5 g or 5 mL of stool specimens were collected at enrollment, before the initiation of any therapy. All specimens were tested at the sentinel hospitals as soon as possible. Otherwise, the stool samples were stored at −80 °C until viral testing or placed in Cary Blair Medium at 4 °C for transporting to the laboratory until bacteria testing. Avoid repeated freezing and thawing of all samples. Following this, all samples were promptly delivered from the sentinel hospitals to their respective district-level Centers for Disease Control and Prevention for standardized testing. To ensure consistency across all district laboratories, standardized operating procedures and reagents are uniformly adopted, and the Beijing Centers for Disease Control and Prevention laboratory conducted regular external quality assessments for all districts laboratories.

Viral diarrhea surveillance samples were tested for rotavirus, norovirus, enterovirus, astrovirus and sapovirus. Viral nucleic acid was extracted. Enteric adenovirus was detected using commercial real-time PCR kit (Bioperfectus Medical Ltd., China), and the others were detected using real-time RT-PCR kits (Bioperfectus Medical Ltd., China), following the manufacturer’s instructions.

Bacterial diarrhea surveillance samples were tested for bacterial pathogens, such as vibrio cholerae, Shigella, *Salmonella typhi* and *Salmonella paratyphi*, DEC, NTS, vibrio parahaemolyticus, campylobacter and others. Bacterial pathogens were detected using culture, biochemical and serological assays.

### 2.3 Estimation of incidence level of diarrhea

The actual incidence of diarrhea was estimated using the Monte Carlo multiplier model. The actual counts of infections were inferred from parameters such as the consultation rate, sampling rate and detection sensitivity.

The multiplier “N” used to scale confirmed cases was defined as follows: The actual number of diarrhea = Laboratory diagnosed diarrhea cases × Multiplier N. Multiplier N = (1/A) × (1/B) × (1/C) = 1/(ABC). “A” specifies the consultation rate of diarrhea, indicating the proportion of cases that required medical consultation at a hospital. For this analysis, parameter A was assigned a value range of 36.9–76.8% (8). “B” specifies the sampling rate, indicating the proportion of cases sampled for laboratory testing. Parameter B was assigned a value range of 3.4–3.9%. “C” is the detection sensitivity, referring to the proportion of positive laboratory test results among cases infected with the pathogen causing diarrhea. Parameter C was assigned a value range of 95.0–100.0%, as per the instructions of the detection reagents.

### 2.4 Statistical analysis

The data were analyzed using SPSS 22.0. The socio-demographic characteristics data and laboratory results were double-entered and validated. Chi-square tests and univariate logistic regression were performed to compare the distributions and detection rates of pathogens between sexes, times, regions, age groups and years. All statistical tests were two-sided, and *p* values <0.05 were considered statistically significant. The average monthly percent change (AMPC) of the main pathogens of diarrhea was analyzed using the Joinpoint Regression Program 4.9.0.0. The multiplicative software model Impact 2009 v 1.0^1^ was used to estimate the true prevalence of diarrhea, which has been commonly adapted and validated for estimating the burden of infectious diseases, including enteric pathogens.

### 2.5 Ethical considerations

This study was approved by the Ethics Committee of Beijing Center for Disease Control and Prevention, and the requirement for informed consent was waived. The study was conducted in accordance with the Declaration of Helsinki (2013 revision). All data were anonymized before analysis, and personal identifiers were removed to ensure participant privacy. All applied methods were carried out in accordance with relevant guidelines and regulations.

## 3 Results

**Figure 1:**
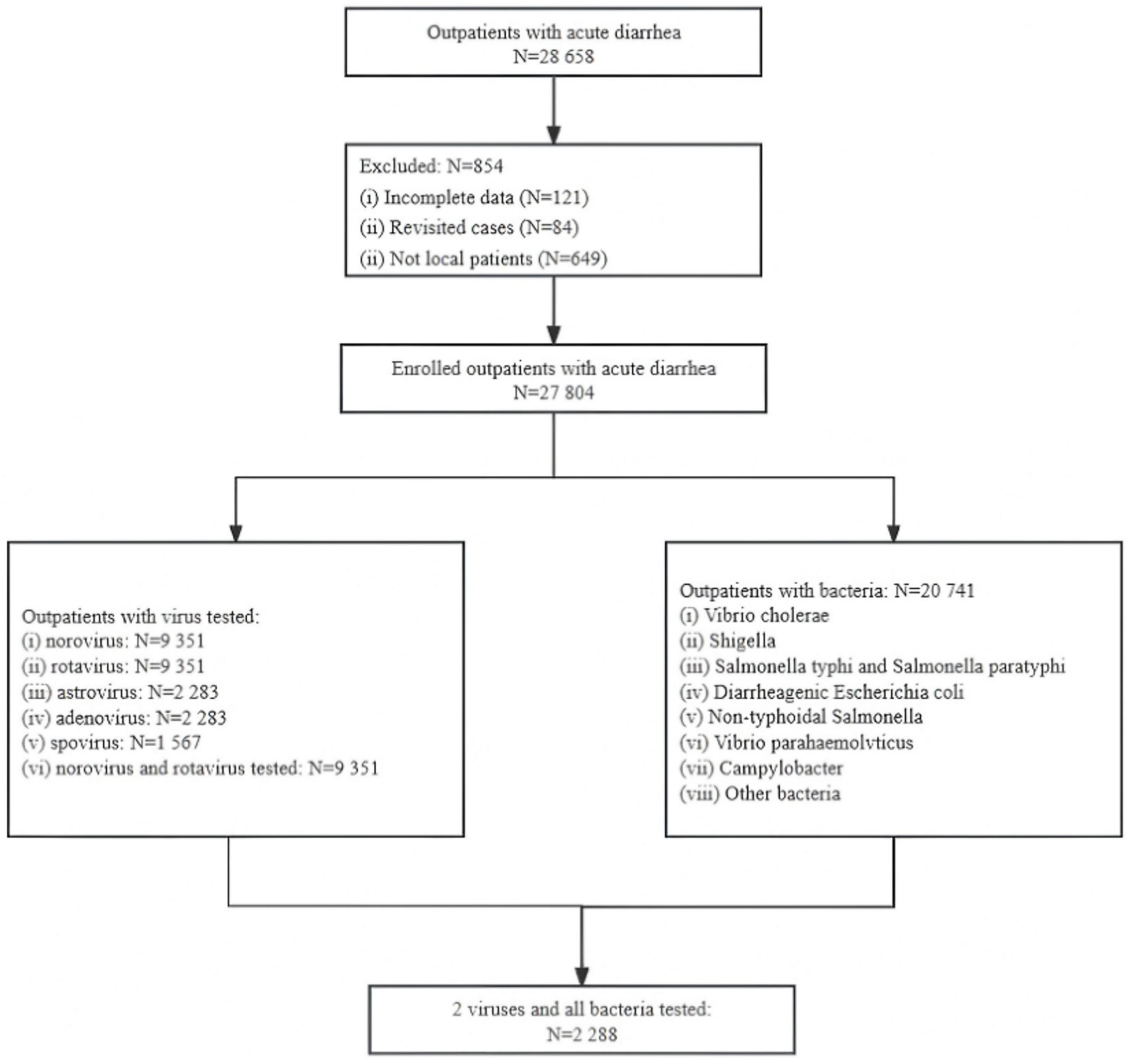
A flowchart of the data collection and sorting procedures.

From 2015 to 2019, 28,658 outpatients with diarrhea were initially included. Eight hundred and fifty-four cases were excluded due to incomplete information (121 cases), revisits (84 cases), and non-local (649 cases). Finally, 27,804 outpatients with diarrhea were included (Figure 1).

Among the included cases, there were 14,905 males and 12,899 females. The cases were mainly young, with a median age of 34 (IQR: 25–54) years old. 11,377 cases (40.92%) were from urban areas, and 16,427 cases (59.08%) were from rural areas (Table 1).

**Table 1:** Demographic characteristics of outpatients with diarrhea in Beijing, China, 2015–2019.

Groups	All cases [*n* (%)] (*N* = 27,804)	Any virus tested [*n* (%)] (*N* = 9,351)	Any bacterium tested [*n* (%)] (*N* = 20,741)	Both viruses and bacteria tested [*n* (%)] (*N* = 2,288)
Sex				
Male	14,905 (53.61)	5,036 (53.86)	11,091 (53.47)	1,222 (53.41)
Female	12,899 (46.39)	4,315 (46.14)	9,650 (46.53)	1,066 (46.59)
Age (years)				
<5	2,389 (8.59)	560 (5.99)	1,905 (9.18)	76 (3.32)
5–17	1,277 (4.59)	434 (4.64)	972 (4.69)	129 (5.64)
18–40	13,349 (48.01)	4,566 (48.83)	9,911 (47.78)	1,128 (49.30)
41–65	7,971 (28.67)	2,809 (30.04)	5,847 (28.19)	685 (29.94)
≥66	2,818 (10.14)	982 (10.5)	2,106 (10.15)	270 (11.80)
Living area				
Urban	11,377 (40.92)	3,468 (37.09)	8,896 (42.89)	987 (43.14)
Rural	16,427 (59.08)	5,883 (62.91)	11,845 (57.11)	1,301 (56.86)
Year				
2015	6,050 (21.76)	2,039 (19.99)	4,147 (21.81)	136 (5.94)
2016	5,460 (19.64)	1,810 (18.23)	3,781 (19.36)	131 (5.73)
2017	5,380 (19.35)	1,781 (17.93)	3,719 (19.05)	120 (5.24)
2018	5,317 (19.12)	1,785 (17.11)	3,549 (19.09)	17 (0.74)
2019	5,597 (20.13)	1,936 (26.73)	5,545 (20.7)	1,884 (82.34)
Season				
Spring	7,303 (26.26)	2,388 (25.53)	5,508 (26.55)	593 (25.91)
Summer	10,421 (37.48)	2,407 (25.74)	8,622 (41.56)	608 (26.57)
Autumn	6,930 (24.92)	2,304 (24.63)	5,205 (25.09)	579 (25.30)
Winter	3,150 (11.32)	2,252 (24.08)	1,406 (6.78)	508 (22.20)

As illustrated in Figure 1, the included cases were tested through two historically independent surveillance streams: a viral pathogen system and a bacterial pathogen system, only a small number of cases were tested for both viruses and bacteria simultaneously. There were 9,351 cases tested for rotavirus and norovirus, and 20,741 cases tested for all bacteria (Figure 1). Among those, 2,288 cases were tested for rotavirus, norovirus and bacteria.

### 3.1 Epidemiological characteristics of patients with viral pathogens

Nine thousand three hundred fifty-one cases were tested for rotavirus and norovirus. Cases were mainly young, and the median age was 35 (IQR: 26–55) years old. 2,283 cases were tested for astrovirus and adenovirus, and 1,567 for sapovirus.

At least one virus was detected in 2,526 (27.01%) cases. Norovirus (1,572/9,351, 16.81%) showed the highest detection rate, followed by rotavirus (907/9,351, 9.70%). The test results for other viruses were as follows: astrovirus (74/2,283, 3.24%), sapovirus (43/1,567, 2.74%), and adenovirus (25/2,283, 1.10%) (Table 2). Among the norovirus-positive cases, 78.20% were infected with GII. A total of 95 cases from the 2,288 patients tested for both virus and bacteria were co-infected with 2 viruses. Among them, norovirus and rotavirus co-infection was mostly detected (70/95, 73.68%), followed by rotavirus and astrovirus co-infection (7/95, 7.37%), and norovirus and astrovirus co-infection (7/95, 7.37%).

**Table 2:** Detection of viral pathogens in outpatients with diarrhea in Beijing, China, 2015–2019.

Groups	Norovirus			Rotavirus		
	Positive rate [*n* (%)]	OR (95% CI)	*P*	Positive rate [*n* (%)]	OR (95%CI)	*P*
Sex						
Male	895/5,036 (17.77)	Reference		458/5,036 (9.09)	Reference	
Female	677/4,315 (15.69)	0.861 (0.772–0.960)	0.007*	449/4,315 (10.41)	1.161 (1.012–1.331)	0.033*
Age (years)						
<5	72/560 (12.86)	Reference		78/560 (13.93)	Reference	
5–17	73/434 (16.82)	1.371 (0.963–1.950)	0.080	20/434 (4.61)	0.299 (0.180–0.496)	0.000*
18–40	880/4,566 (19.27)	1.618 (1.250–2.095)	0.000*	333/4,566 (7.29)	0.486 (0.373–0.633)	0.000*
41–65	439/2,809 (15.63)	1.255 (0.961–1.641)	0.096	379/2,809 (13.49)	0.964 (0.741–1.253)	0.783
≥66	108/982 (11.00)	0.838 (0.609–1.151)	0.275	97/982 (9.88)	0.677 (0.493–0.931)	0.016
Living area						
Urban	655/3,468 (18.89)	Reference		296/3,468 (8.54)	Reference	
Rural	917/5,883 (15.59)	0.793 (0.710–0.886)	0.000*	611/5,883 (10.39)	1.242 (1.074–1.437)	0.004*
Year						
2015	404/2,039 (19.81)	Reference		175/2,039 (8.58)	Reference	
2016	264/1,810 (14.59)	0.691 (0.583–0.819)	0.000*	204/1,810 (11.27)	1.353 (1.094–1.674)	0.005*
2017	327/1,781 (18.36)	0.910 (0.774–1.070)	0.255	205/1,781 (11.51)	1.385 (1.12–1.714)	0.003*
2018	291/1,785 (16.30)	0.788 (0.668–0.931)	0.005*	179/1,785 (10.03)	1.187 (0.954–1.478)	0.124
2019	286/1,936 (14.77)	0.701 (0.594–0.828)	0.000*	144/1,936 (7.44)	0.856 (0.680–1.077)	0.185
Season						
Spring	580/2,388 (24.29)	Reference		267/2,388 (11.18)	Reference	
Summer	283/2,407 (11.76)	0.415 (0.356–0.485)	0.000*	20/2,407 (0.83)	0.067 (0.042–0.105)	0.000*
Autumn	310/2,304 (13.45)	0.485 (0.416–0.564)	0.000*	60/2,304 (2.60)	0.212 (0.160–0.283)	0.000*
Winter	399/2,252 (17.72)	0.671 (0.582–0.774)	0.000*	560/2,252 (24.87)	2.629 (2.242–3.083)	0.000*
Total	1,572/9,351 (16.81)			907/9,351 (9.70)		

* indicates statistically significant differences (*p* < 0.05).

Compared with male patients, female patients had lower risk odds for norovirus (*OR* = 0.861, 95% *CI*: 0.772–0.960), but higher risk odds for rotavirus (*OR* = 1.161, 95% *CI*: 1.012–1.331). People who lived in rural areas were less likely to be norovirus-positive (*OR* = 0.793, 95% *CI*: 0.710–0.886), while more likely to be rotavirus-positive (*OR* = 1.242, 95% *CI*: 1.074–1.437). The viral pathogen spectrum differed across age groups and years. Adults aged 18–40 years old had the highest norovirus-positive rate (19.27%), while the highest rotavirus-positive rate was detected in children under 5 years of age (13.93%). Norovirus-positive rate was the highest in 2015 (19.81%), but rotavirus-positive rate was the highest in 2017 (11.51%). The positive rates of norovirus and rotavirus were both higher in winter and spring.

**Figure 2:**
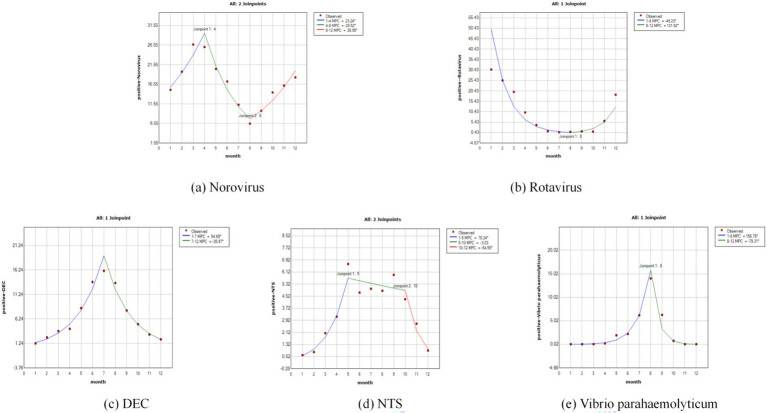
The joinpoint regression of the positive rates of enteropathogens by months: **(a)** Norovirus; **(b)** Rotavirus; **(c)** DEC; **(d)** NTS; **(e)** *Vibrio parahaemolyticus*.

Norovirus exhibited a distinct seasonal pattern, with a primary peak in the winter and spring months and a marked trough during the summer. Joinpoint regression showed that norovirus-positive rates significantly increased from January to April (MPC_1–4_ = 23.24, 95% *CI* = 3.21–47.16%, *p* = 0.031), decreased from April to August (MPC_4–8_ = −28.52, 95% *CI* = −40.14% to −14.64%, *p* = 0.006) and obviously increased from August to December (MPC_8–12_ = 26.95, 95% *CI* = 13.48–42.02%, *p* = 0.004) (Figure 2a). Rotavirus activity demonstrated a characteristic pattern, with low activity throughout the summer followed by a sharp increase beginning in the autumn. While rotavirus showed a significantly decreasing trend from January to August (MPC_1–8_ = −49.23, 95% *CI* = −59.29% to −36.69%, *p* = 0.031), and an increasing trend from August to December (MPC_8–12_ = 131.92, 95% *CI* = 37.54–291.07%, *p* = 0.007) (Figure 2b).

### 3.2 Epidemiological characteristics of patients with bacterial pathogens

Twenty thousand seven hundred forty-one cases were tested for bacterial pathogens. The cases were mainly young, and the median age was 34 (IQR: 25–54) years old.

At least one bacterium was detected in 4,455 (21.48%) cases. DEC (9.36%, 1,941/20,741) showed the highest detection rate, followed by NTS (4.55%, 944/20,741) and vibrio parahaemolyticus (4.16%, 864/20,741) (Table 3). Notably, the positive rate of campylobacter has increased rapidly in recent years. A total of 270 cases were co-infected with 2 or 3 bacteria. Among them, DEC and vibrio parahaemolyticus co-infection was mostly detected (62/270, 22.96%), followed by DEC and NTS co-infection (35/270, 12.96%). The extraordinarily wide confidence intervals for some estimates (e.g., campylobacter) reflect statistical uncertainty due to low event counts in comparison groups.

**Table 3:** Detection of bacterial pathogens in outpatients with diarrhea in Beijing, China, 2015–2019.

Groups	DEC			NTS			Vibrio parahaemolyticum			Campylobacter		
	Positive rate [*n*/*N*, %]	OR (95% CI)	*P*	Positive rate [*n*/*N*, %]	OR (95% CI)	*P*	Positive rate [*n*/*N*, %]	OR (95% CI)	*P*	Positive rate [*n*/*N*, %]	OR (95% CI)	*P*
Sex												
Male	1,062/11,091 (9.58)	Reference		509/11,091 (4.58)	Reference		448/11,091 (4.03)	Reference		264/11,091 (2.38)	Reference	
Female	879/9,650 (9.11)	0.946 (0.862–1.040)	0.250	435/9,650 (4.50)	0.981 (0.861–1.119)	0.779	416/9,650 (4.31)	1.070 (0.934–1.227)	0.329	176/9,650 (1.82)	0.762 (0.628–0.924)	0.006*
Age (years)												
<5	159/1,905 (8.35)	Reference		114/1,905 (5.98)	Reference		5/1,905 (0.26)	Reference		3/1,905 (0.15)	Reference	
5–17	78/972 (8.02)	0.958 (0.722–1.271)	0.767	46/972 (4.73)	0.780 (0.549–1.109)	0.167	19/972 (1.95)	7.576 (2.820–20.352)	0.000*	20/972 (2.05)	13.319 (3.948–44.934)	0.000*
18–40	1,021/9,911 (10.30)	1.261 (1.059–1.502)	0.009*	375/9,911 (3.78)	0.618 (0.498–0.766)	0.000*	573/9,911 (5.78)	23.318 (9.655–56.313)	0.000*	266/9,911 (2.68)	17.485 (5.598–54.618)	0.000*
41–65	511/5,847 (8.74)	1.052 (0.873–1.267)	0.596	303/5,847 (5.18)	0.859 (0.688–1.072)	0.178	225/5,847 (3.84)	15.208 (6.259–36.950)	0.000*	112/5,847 (1.91)	12.382 (3.929–39.018)	0.000*
≥66	172/2,106 (8.17)	0.977 (0.780–1.223)	0.837	106/2,106 (5.03)	0.833 (0.634–1.093)	0.187	42/2,106 (1.99)	7.733 (3.053–19.585)	0.000*	39/2,106 (1.85)	11.962 (3.691–38.772)	0.000*
Living area												
Urban	793/8,896 (8.91)	Reference		405/8,896 (4.55)	Reference		306/8,896 (3.43)	Reference		197/8,896 (2.21)	Reference	
Rural	1,148/11,845 (9.69)	1.097 (0.997–1.206)	0.057	539/11,845 (4.55)	1.000 (0.876–1.140)	0.994	558/11,845 (4.71)	1.388 (1.204–1.600)	0.000*	243/11,845 (2.05)	0.925 (0.765–1.118)	0.420
Year												
2015	251/4,147 (6.05)	Reference		165/4,147 (3.97)	Reference		106/4,147 (2.55)	Reference		0/4,147 (0.00)	-	-
2016	351/3,781 (9.28)	1.588 (1.342–1.880)	0.000*	156/3,781 (4.12)	1.039 (0.831–1.299)	0.740	265/3,781 (7.00)	2.873 (2.284–3.615)	0.000*	3/3,781 (0.07)	Reference	
2017	336/3,719 (9.03)	1.542 (1.301–1.827)	0.000*	149/3,719 (4.00)	1.007 (0.803–1.263)	0.950	149/3,719 (4.00)	1.591 (1.235–2.049)	0.000*	14/3,719 (0.37)	4.759 (1.366–16.572)	0.014*
2018	389/3,549 (10.96)	1.911 (1.619–2.255)	0.000*	214/3,549 (6.02)	1.549 (1.258–1.907)	0.000*	156/3,549 (4.39)	1.753 (1.364–2.253)	0.000*	116/3,549 (3.26)	42.552 (13.513–133.994)	0.000*
2019	614/5,545 (11.07)	1.933 (1.659–2.252)	0.000*	260/5,545 (4.68)	1.187 (0.973–1.449)	0.092	188/5,545 (3.39)	1.338 (1.051–1.703)	0.018*	307/5,545 (5.53)	73.810 (23.656–230.295)	0.000*
Season												
Spring	338/5,508 (6.14)	Reference		257/5,508 (4.66)	Reference		54/5,508 (0.98)	Reference		98/5,508 (1.77)	Reference	
Summer	1,251/8,622 (14.51)	2.596 (2.290–2.943)	0.000*	421/8,622 (4.88)	1.049 (0.895–1.230)	0.556	642/8,622 (7.44)	8.126 (6.142–10.749)	0.000*	192/8,622 (2.22)	1.177 (0.911–1.521)	0.213
Autumn	325/5,205 (6.24)	1.019 (0.870–1.192)	0.818	255/5,205 (4.89)	1.053 (0.881–1.257)	0.572	168/5,205 (3.22)	3.369 (2.473–4.588)	0.000*	140/5,205 (2.68)	1.568 (1.199–2.049)	0.001*
Winter	27/1,406 (1.92)	0.299 (0.201–0.445)	0.000*	11/1,406 (0.78)	0.161 (0.088–0.295)	0.000*	0/1,406 (0.00)	0 (0)	0.988	10/1,406 (0.71)	0.422 (0.219–0.812)	0.010*
Total	1,941/20,741 (9.36)			944/20,741 (4.55)			864/20,741 (4.16)			440/20,741 (2.12)		

* indicates statistically significant differences (*p* < 0.05).

Among the 1,975 DEC-positive cases, enteroaggregative *E. coli* was most frequently detected (35.65%), followed by enterotoxigenic *E. coli* (34.53%), enteropathogenic *E. coli* (27.95%), enteroinvasive *E. coli* (0.96%), and enterohaemorrhagic *E. coli* (0.91%). Thirty-four cases were detected positive for two serologic DECs.

The positive rates of DEC and vibrio parahaemolyticus were both the highest in adults aged 18–40 years old, while that of NTS was in children under 5 years of age. People who lived in rural areas had a higher risk for vibrio parahaemolyticus (*OR* = 1.388, 95% *CI*: 1.204–1.600). The bacterial pathogen spectrum differed across years. The positive rates of DEC and campylobacter increased year by year. The detection rate of NTS was the highest in 2018, while vibrio parahaemolyticus was the highest in 2016. The positive rates of main bacterial pathogens were higher in the summer.

DEC displayed a clear summer seasonality, with detection rates rising sharply in the warmer months to a single peak. Joinpoint regression showed that the DEC-positive rate significantly increased from January to July (MPC_1–7_ = 54.69, 95% *CI* = 44.55–65.54%, *p* < 0.001), and obviously decreased from July to December (MPC_7–12_ = −35.97, 95% *CI* = −41.47% to −29.97%, *p* < 0.001) (Figure 2c). NTS showed elevated detection rates from late spring through autumn, indicating warm-season predominance. The positive rate of NTS showed a significantly increasing trend from January to May (MPC_1–5_ = 78.24, 95% *CI* = 49.38–112.69%, *p* = 0.001) and a clearly decreasing trend from October to December (MPC_10–12_ = −54.55, 95% *CI* = −74.00% to −20.53%, *p* = 0.017) (Figure 2d). *Vibrio parahaemolyticus* was strongly seasonal, with a sharp peak in the mid-summer months. The monthly epidemic pattern of vibrio parahaemolyticus was basically the same as DEC, except that the turning point was in August (MPC_1–8_ = 156.78, 95% *CI* = 80.98–264.31%, *p* < 0.001; MPC_8–12_ = −79.31, 95% *CI* = −90.96% to −52.67%, *p* = 0.003) (Figure 2e).

### 3.3 Epidemiological characteristics of patients with both viral and bacterial pathogens tested

To explore pathogen co-infections, we analyzed the cases where both viral and bacterial pathogens were detected simultaneously (*N* = 2,288). The majority of cases (1,884) were from 2019. Viral pathogens were detected positively in 584 cases (25.52%), higher than bacterial pathogens (19.76%). The pathogen with the highest positive rate was norovirus (16.81%), followed by rotavirus (9.70%), DEC (8.87%), campylobacter (4.46%) and NTS (4.11%). One hundred seven cases from the 2,288 tested for both viruses and bacteria tested positive for 2 or more pathogens. Among them, 62 cases were virus and bacterium co-infection. Norovirus and DEC co-infection was mostly detected (23/107, 21.50%). Twenty-one cases were co-infected with 2 bacteria, 22 cases were co-infected with 2 viruses. One case was co-infected with DEC, vibrio parahaemolyticus and norovirus, and 1 case was co-infected with norovirus, DEC and sapovirus. Given that this integrated analysis was limited in scale and primarily reflective of a single year, these generate reference for future research.

**Figure 3:**
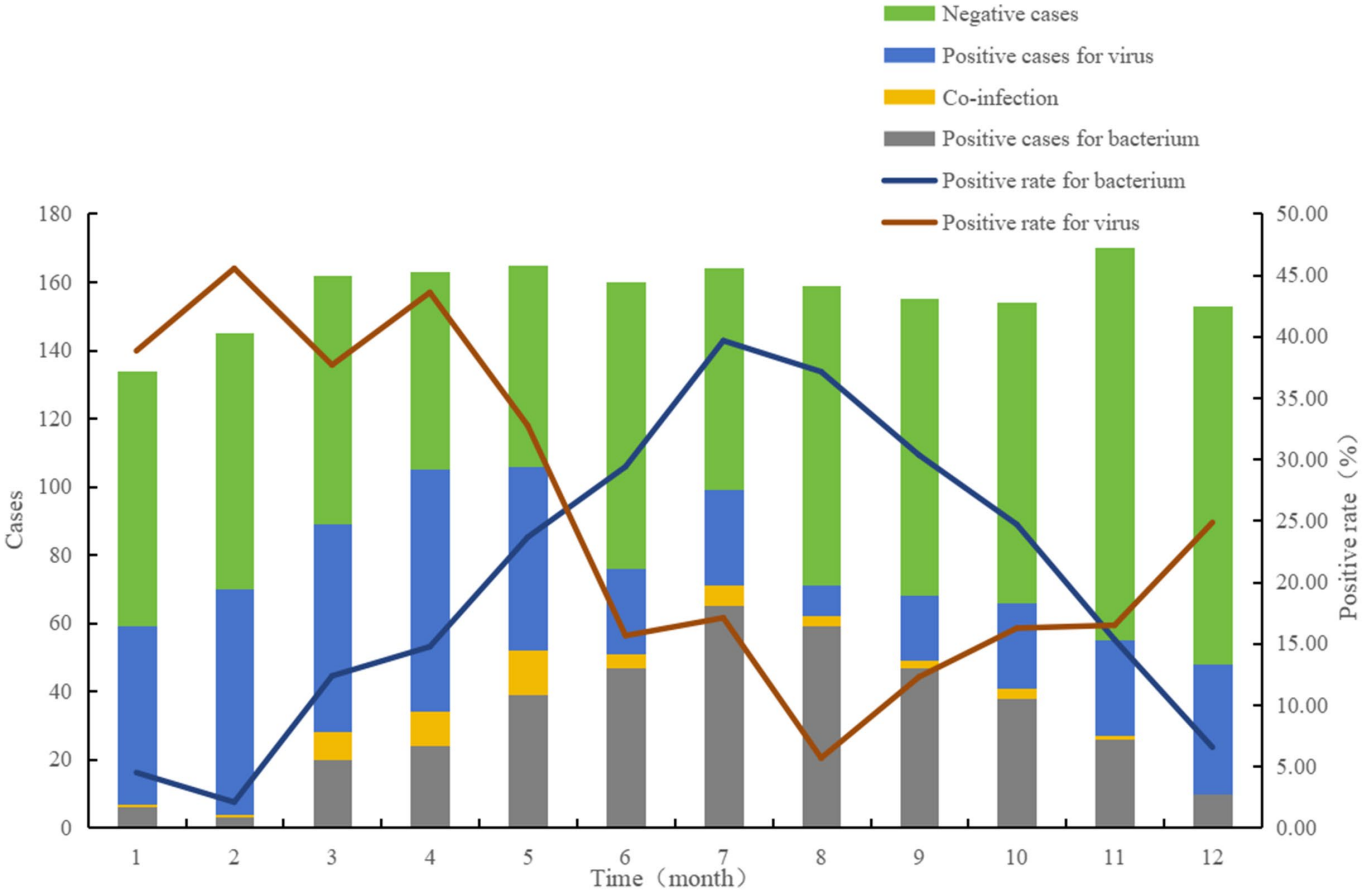
Positive rates and counts of enteropathogens in outpatients with diarrhea in Beijing, China, 2019.

The monthly positive rates for bacteria and viruses in 2019 were shown in Figure 3. The positive rate for bacterial pathogens was increasing since February, reaching the highest in July (39.63%). The positive rate for viral pathogens showed a different pattern, with the highest rate in February (45.52%).

### 3.4 Estimation of incidence level of infectious diarrhea

**Figure 4:**
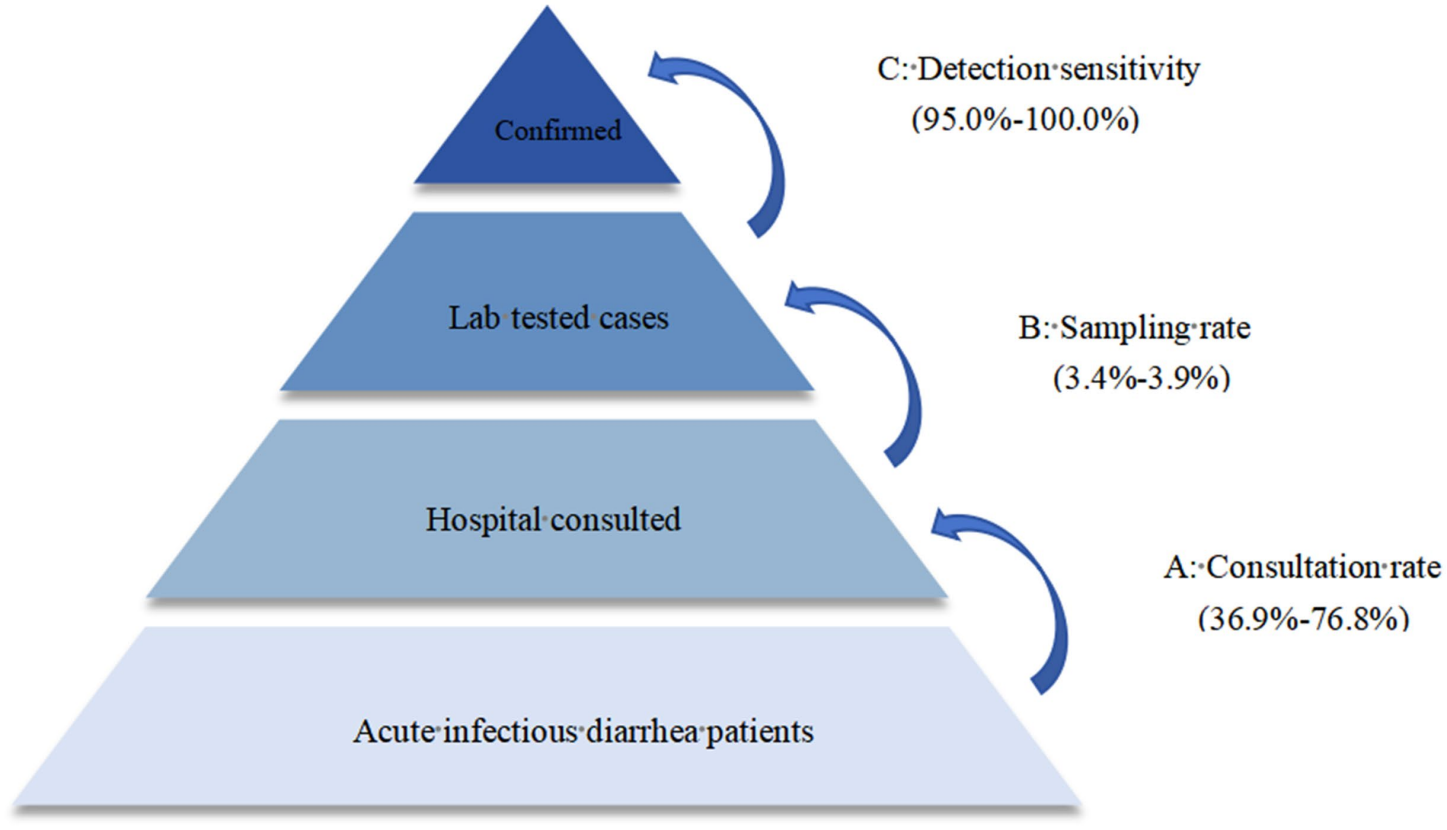
Parameters for the estimation of actual infections of diarrhea using the multiplier model.

The reference range selected for the consultation rate (A) was 36.9–76.8% (8). The selected range for the sampling rate (B) was 3.4–3.9%. According to the instructions of the detection reagents, the sensitivity (C) was selected within the range of 95.0–100.0% (Figure 4).

Estimated by the model, the average annual number of infectious diarrhea cases in Beijing from 2015 to 2019 was approximately 68,054 (95% *CI*: 49,917–104,149). There were 314 (95% *CI*: 231–481) infectious diarrhea cases per 100,000 people. One base case actually represents 49 cases.

## 4 Discussion

Diarrhea has a high disease burden worldwide, but the predominant pathogens in different regions vary. Viral pathogens are predominant in developed countries (9) and bacterial pathogens in developing countries (10). Both viruses and bacteria are prevalent in China owing to its vastness (7). Beijing has established surveillance networks of viral and bacterial pathogens for patients with diarrhea, which are constantly improving. Due to the differing epidemiological characteristics of viruses and bacteria, the monthly sampling volumes for the two differ. This study analyzed data from the period when both surveillance networks were carried out in 16 districts until the COVID-19 pandemic. The data were relatively stable during this period. To ensure data consistency across the 2015–2019 study period, the following analysis of pathogen detection rates is based on the primary viral and bacterial surveillance systems. Since the integrated surveillance system, which is designed for the combined detection of viruses and bacteria, was not widely implemented on a large scale until 2019, the data from the established separate systems offer a more representative overview for the entire duration.

A nationwide study found that the detection rate of bacterial pathogens (18.23%) was lower than that of viral pathogens (31.86%) in China (7). This study also found that the detection rate of viral pathogens (27.01%) was higher than bacterial pathogens (21.48%), which is in line with the trend observed in the nationwide study. The different detection rates of viral and bacterial pathogens might be related to the different detection methods. Additionally, other factors such as the age distribution of the study population could also be potential confounders. The prevalence of viruses and bacteria showed different seasonal trends. Overall, the detection rate of viral pathogens was higher in winter and spring, and that of bacterial pathogens was higher in summer and autumn (11). Notably, the study by Fang et al. (12) further demonstrated that these trends were age-specific, with viruses predominating in younger children (0–5 years) during winter and bacteria being more associated with adult cases (>20 years) in summer. There was definite evidence that meteorological factors affected intestinal infectious diseases (13–15). High temperature might enhance bacterial survival and growth in reservoirs and food sources, and increase the risk of bacterial diarrhea (16). In terms of pathogens, DEC and *Vibrio parahaemolyticus* both showed monthly peaks, concentrated in July or August. The detection rate of NTS remained high from May to October. Outdoor dining activities in summer and autumn are also the main reason for the high incidence of bacterial diarrhea (17). Viral pathogens such as norovirus and rotavirus are adapted to low temperatures (7). Norovirus detection rate increased in winter and spring, while rotavirus increased in autumn and winter, both being lowest in August. Crowded places without adequate ventilation, especially in schools, may promote the occurrence and spread of viral diarrhea (18). In this study, the positive rate of norovirus was higher in urban areas. Urban areas exhibit higher population density, greater reliance on public transportation, and more large congregate settings compared to rural areas, all of which contribute to an increased risk of norovirus transmission (19).

We used Joinpoint regression to identify nonlinear trends and inflection points in the seasonal variations of positive rates for five major enteric pathogens (7). Norovirus exhibited a characteristic pattern of “winter peak and summer trough.” A significant inflection point was identified for rotavirus in August, which may be associated with the accumulation of susceptible populations (20). DEC and vibrio parahaemolyticus demonstrated similar trends, while NTS displayed a relatively stable temporal pattern. These findings provide crucial insights into the epidemic dynamics of both viral and bacterial pathogens, offering practical significance for determining key time windows for implementing public health interventions. However, it is important to note that this analytical approach may carry a risk of overfitting (21).

In recent years, acute gastroenteritis caused by norovirus has attracted much attention. A meta-analysis on norovirus acute gastroenteritis prevalence found that norovirus infection accounted for about 20% of acute gastroenteritis worldwide (9). The proportion in developed countries is higher than 20% (22, 23), while about 17% in developing countries (24, 25). Norovirus is the most common pathogen detected among adults. However, rotavirus is a leading cause of morbidity and mortality in children under 5 years of age worldwide, especially in developing countries (25). This study showed similar results, with the rotavirus detection rate being highest in children under 5 years of age while the norovirus detection rate being highest in the group aged 18–40 years old. Meanwhile, the rotavirus detection rate in children under 5 years of age in 2019 was lower than in our previous study (13.93% vs. 15.61) (26), which might be related to the introduction of the live, oral pentavalent rotavirus vaccine (RotaTeq) into Beijing in 2019, and its coverage has since increased (20). Research indicates that since the implementation of the RotaTeq vaccine, the RVA-positive rates among children under five and adults with acute diarrhea in 2019 significantly decreased to 9.45 and 3.66%, respectively (20).

DEC was the highest positive rate of bacterial pathogens, followed by NTS and vibrio parahaemolyticus, consistent with the result of the nationwide study in China (7). A key finding of our study was the distinctive profile of DEC serotypes in Beijing, with EAEC being the most prevalent (35.65%). This contrasts with reports from other regions, such as southeastern China and among travelers, where EAEC is often dominant (27, 28). This highlights the unique local characteristics of diarrheal etiology in Beijing, which may be influenced by regional socioeconomic and geographical factors (10, 29, 30). The predominance of EAEC, a pathogen known for its role in both acute and persistent diarrhea, suggests a need for enhanced monitoring and prevention efforts by local public health authorities. In coastal areas, due to seafood exposure and consumption, the detection rate of vibrio parahaemolyticus was higher than NTS and DEC (31), while Beijing belongs to non-coastal areas. These results emphasize the necessity for developing region-specific public health strategies and surveillance systems that account for local pathogen profiles and risk factors.

Compared with other age groups, DEC, vibrio parahaemolyticus and campylobacter were all at a higher risk of testing positive in adults aged 18 to 40 years old, which may be related to young people being more exposed to infections. This condition may be attributed to broader exposure opportunities common in this people, including high-frequency dining out, consumption of ready-to-eat foods, and elevated occupational mobility. It is recommended to conduct health education on food safety and personal hygiene while strengthening oversight of street food vendors and takeout services, which would help reduce transmission risks among this high-risk population.

The cases of vibrio cholerae in Beijing were highly sporadic. Both the detection rate of Shigella and its proportion of bacterial pathogens are gradually declining, which may be related to the advancement of urbanization and public health awareness in China. However, imported cases need to be noted, as traveling to countries where cholera or dysentery is endemic might lead to local epidemics and outbreaks.

Our incidence estimation provides a valuable measure of the total burden of infectious diarrhea. The model estimates an annual average of 68,054 cases, implying a ratio of approximately 49 actual cases for every one laboratory-confirmed case reported. However, this finding is not a failure of surveillance but as a quantifiable outcome of its current operational context, which includes the public’s healthcare-seeking behavior and selective pathogen testing due to resource constraints. This estimated burden is consistent with a nationwide modeling study in China (7). The high under-ascertainment ratio implies that surveillance data alone may substantially underestimate the true transmission dynamics and total burden. Therefore, in future work, we recommend a multi-pronged approach: first, conducting targeted community surveys to better understand healthcare access and testing barriers; second, ensuring that primary care and community health centers are accessible and equipped to manage diarrheal cases promptly; third, developing and promoting the use of rapid, affordable diagnostic test kits that could be used at home or in primary care settings to facilitate early pathogen identification and appropriate treatment; and finally, strengthening public health education to promote good hygiene practices and encourage timely medical consultation when symptoms become severe. These integrated measures would enhance case detection, improve individual outcomes, and provide a more accurate assessment of the true disease burden while maintaining the stability and utility of the existing surveillance system for tracking long-term trends.

From 2015 to 2019, an annual average of approximately 39,000 cases of infectious diarrhea were reported in the Beijing hospital network, which was only a part of the actual number of infections. Estimating the actual number of diarrhea cases in Beijing is necessary, as it contributes to a comprehensive understanding of the social burden caused by diarrhea and formulates reasonable plans or strategies. The multiplicative model based on the Monte Carlo had been applied to estimate the actual number of people infected with various diseases (32). However, monitoring data may be subject to multiple potential underestimation factors. Such as, many mild cases may not seek medical care, and even among those who do visit healthcare facilities, not all patients testing for intestinal pathogens. Furthermore, variations in diagnostic test sensitivity may also impact detection rates. After probabilistically adjusting for these factors, results indicate that the actual burden of diarrheal diseases in Beijing may be underestimated. Our findings identify adults aged 18–40 years as a high-risk group, with the highest positive rates for key pathogens including norovirus, DEC, and campylobacter. This trend may be driven by high-density working environments and frequent social or business dining. While our surveillance did not capture individual behavioral exposures, the pattern aligns with findings from other studies that associate these factors with increased infection risk in young adults (19). These findings indicate the necessity of continuously strengthening systematic surveillance to gain a more comprehensive understanding of the pathogen’s impact on the population, thereby facilitating early detection and enabling timely, targeted public health response measures (33).

There are some limitations to this study. First, the collection of cases was not random, and there may be some bias in the detection rate of virus. While this sampling method is convenient to implement in practical surveillance and ensures consistent data across districts and seasons, statistically speaking, the sample size should be dynamically adjusted on a quarterly or monthly basis according to the prevalence of pathogens. The current practice of evenly distributing the monthly sampling quota across weeks at each surveillance site—where approximately one-quarter of the monthly target is collected per week, with shortfalls compensated in subsequent weeks—aims to reduce temporal clustering bias. However, it does not fully overcome the inherent representativeness constraints of non-random sampling. This uniform sampling pattern may still lead to an overestimation of viral prevalence throughout the year, particularly if there is spatial or temporal heterogeneity in pathogen circulation (34). Future surveillance programs should be further refined, for example, dynamically adjusting sampling quotas based on real-time epidemiological data. Second, due to differences in testing capacity and financial support at district laboratories, not all enrolled diarrhea outpatients were tested for all listed pathogens. Third, the pathogens tested were limited, and negative cases may be infected with untested pathogens. Furthermore, although the multiplicative model based on Monte Carlo simulation helps estimate the underreported burden of diarrheal disease, it relies on assumptions regarding healthcare-seeking behavior and test sensitivity that may affect accuracy. Despite these limitations, the findings still reveal a substantial diarrheal disease burden in Beijing, underscoring the need for sustained and refined surveillance.

## 5 Conclusion

Diarrhea remains a serious public health problem in Beijing, China. Viral and bacterial pathogens alternate in prevalence throughout the year. Norovirus, DEC and rotavirus were the main pathogens among diarrhea outpatients in Beijing before the COVID-19 pandemic. Continuous epidemiological and etiological surveillance is necessary for the diagnosis and treatment of diarrhea and the implementation of targeted prevention and control measures.

## Data Availability

The raw data supporting the conclusions of this article will be made available by the authors, without undue reservation.

## Footnotes

1
https://www.cdc.gov/h1n1flu/tools/impact2009/index.htm
